# Supplementary material for: ArGSLab: a tool for analyzing experimental or simulated particle networks
Source: Soft Matter. 2021 Aug 24;17(36):8354–62. doi: 10.1039/d1sm00692d (PMC8457054; doi:10.1039/d1sm00692d)
Supplement: SM-017-D1SM00692D-s001 [file SM-017-D1SM00692D-s001.pdf]

Electronic Supplementary Material (ESI) for Soft Matter.
This journal is © The Royal Society of Chemistry 2021

Illustration of ArGSLab analysis parameters

Version 1.0

July 30, 2021

To help familiarize users with the functions and parameters of ArGSLab, we have added an example image stack to the ArGSLab repository that is intended for allowing users to accustom themselves to ArGSLab's parameters, as well as illustrating certain parameters directly. This image stack is an idealized, computer-generated image for illustrating the effect of the various analysis parameters. In this section, we will show how to analyze this image stack using ArGSLab, and in which way certain parameters affect the final result. Several slices of this image stack are depicted in Figure 1. The image stack is 320x320x320 pixels, with a grid of 5x5x5 straight lines. At z = 90, the grid is branched with offshoots of various length (Figure 1B); at z $\sim$ 160, the grid is branched such that it creates a tangled network around a central node (Figure 1C); and at z = 230 and z = 265, the network is interrupted in x, y and z with gaps of various lengths (Figure 1D).

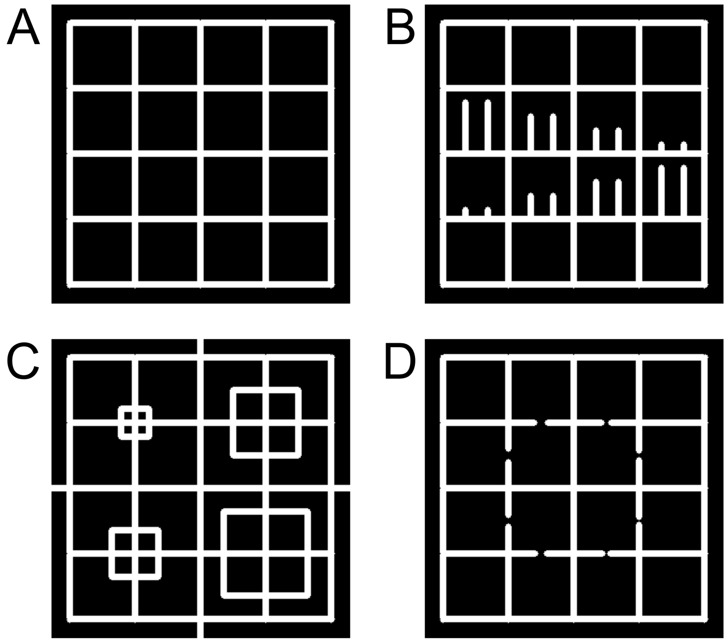

Figure 1: Slices from the raw benchmarking image, as obtainable from the ArGSLab repository. A) Slice at z = 20. B) Slice at z = 90, including the branches of various lengths. C) Slice at z = 160, with tangled node networks. D) Slice at z = 230, with gaps interrupting the grid at various points.

ArGSLab can be initialized by putting the example image stack in a folder and, if one uses ArGSLab's standalone version, set `projectpath` as the path from "main.m" to the folder containing the image stack. If using the GUI version, clicking "Project folder" will prompt a browser pop-up where this

Table 1: Default parameters for ArGSLab.

Parameter	Value	Unit
sigmablur	2	voxels
binThres	'auto'	
sesize	[0.5,0.5,0.5]	σ
minfrac	0.001	
h_fill_thr	1	σ
term_skel_thr	1.5	σ
coll_skel_thr	0.9	σ
edgenode_dist	1	σ

folder should be clicked. Then, if the user is satisfied with the parameters (default parameters can be found in Table 1), "main.m" can be run (Standalone version) or "Run full analysis" can be clicked (GUI version). Results will appear in an "Output" folder in the user-specified folder.
As ArGSLab is intended for particulate matter gels, it requires the length of an input voxel in each dimension, and the diameter of a particle in each dimension. The particle diameter σ is used to define length scales for other parameters. For the purposes of this example, we set the voxel sizes to be [1,1,1] (μm) and the particle diameter to be 10 (μm).

Using default settings for all parameters, we obtain the image output as seen in Figure 2. The three parameters which we will discuss in this walkthrough are the following: `term_skel_thr`, `coll_skel_thr`, and `sesize`. `term_skel_thr` governs the minimum length of terminal branches to be kept (more information below, most detailed information in Manual section 5.2); `coll_skel_thr` governs to what extent clusters of proximate nodes are collected (more information below, most detailed information in Manual section 5.2); and `sesize` governs the size of the size of the morphological closing structural element (more information below and in the Article). In the following paragraphs, we will show the output while varying these three parameters, while keeping all other parameters as their default values.

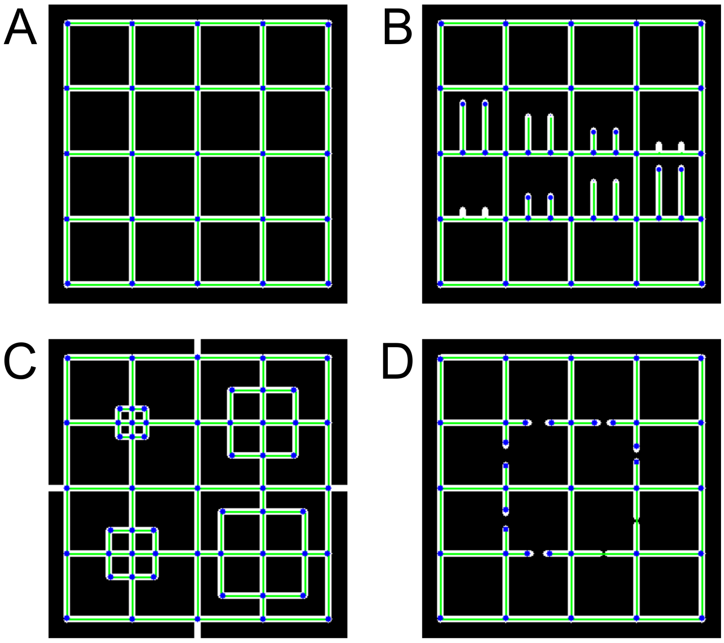

Figure 2: Slices from the analyzed image, from the outputted binarized_skeleton image: the skeleton overlaid over the binarized image. The skeleton is in green, the nodes in blue. The parameters were the preset default parameters. A) Slice at z = 20. B) Slice at z = 90, including the branches of various lengths. Note that the shortest lengths are removed. C) Slice at z = 160. D) Slice at z = 230. Note that the shortest gap is removed by the default settings.

term_skel_thr

In Figure 3, the raw accustomization image is depicted four times, each overlaid with the skeleton and nodes generated with a different value of term_skel_thr. This image is called "rawimage_skeleton.tif" in the output. The images shown are the slices at z = 90. The values of term_skel_thr are 1.5, 3.0, 4.5 and 6.0 ([σ]) for Figure 3A, B, C and D respectively. The remainder of the parameters are the default values as seen in Table 1.

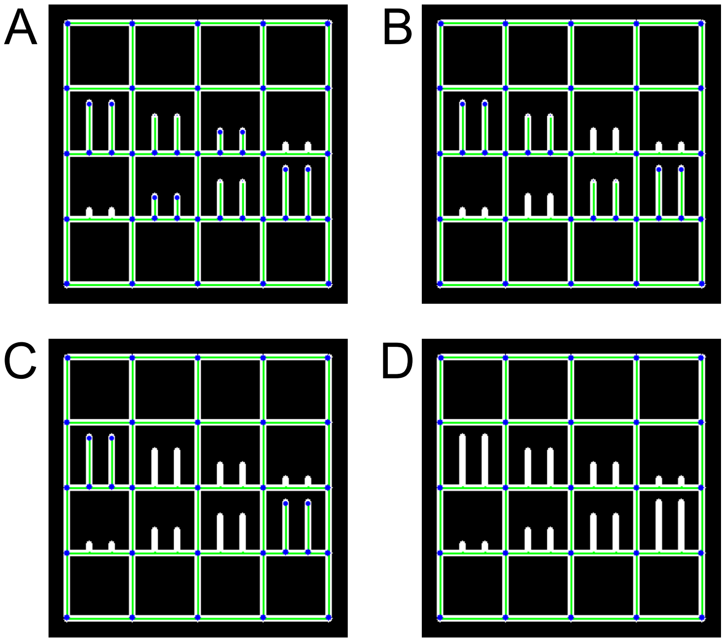

Figure 3: Overlays of ArGSLab's skeleton over the raw benchmarking image, using a different term_skel_thr but all other parameters default and constant. A) term_skel_thr = 1.5, B) term_skel_thr = 3.0, C) term_skel_thr = 4.5, D) term_skel_thr = 6.0.

It is clearly seen that increasing term_skel_thr prunes terminal side branches if they are below a certain threshold length. The maximum length in units of σ is defined by term_skel_thr, and the length of σ is defined by the set voxel sizes and particle diameter. Using our settings, σ is 10 voxel lengths in each dimension. This means that in our example where term_skel_thr = 3.0, any terminal link with a length of 30 voxels or shorter will be pruned. It is important to mention that, if terminal side branches possess their own side branches, these might be pruned as well. How these are pruned exactly is discussed and shown in Section 5.2 and Figure 9 of the Manual.

coll_skel_thr

In Figure 4, the raw accustomization image is depicted four times, each overlaid with the skeleton and nodes generated with a different value of `coll_skel_thr`. The images shown are all at z = 160. The values are 0.9, 1.8, 2.7 and 3.6 σ for Figure 4A, B, C and D respectively. The remainder of the parameters are the default values.

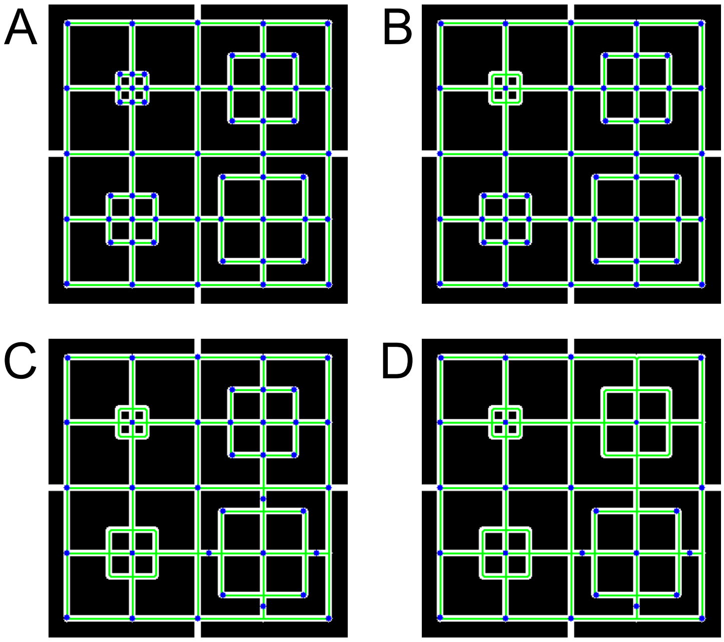

Figure 4: Overlays of ArGSLab's skeleton over the raw benchamrking image, using a different `coll_skel_thr` but all other parameters default and constant. A) `coll_skel_thr` = 0.9 σ, B) `coll_skel_thr` = 1.8 σ, C) `coll_skel_thr` = 2.7 σ, D) `coll_skel_thr` = 3.6 σ.

It can be seen that increasing `coll_skel_thr` leads to nodes that are more and more distant getting collected into a central node. This subroutine functions as follows. It first divides all nodes into certain sets, where each node in a set is at maximum `coll_skel_thr` σ away (along the skeleton) from another node of that set. All nodes of these sets are subsequently collected into one central node. This central node position is calculated by obtaining the center of mass of all nodes in the set, and finding the voxel on the skeleton

that is closest to that center of mass in terms of Euclidean distance. Finally, all nodes are removed, only the central node remaining, and all links are connected and their lengths updated. `coll_skel_thr` removes multitudes of nodes in close proximity to each other, which is often found in images with dense local clusters, exacerbated by noisy data. However, a too large `coll_skel_thr` removes nodes unnecessarily, as seen in Figure 4D: nodes in the bottom right quadrant are collected in the node in the top right quadrant, with other nodes being unnecessarily connected as well. A careful tuning of this parameter is recommended, with the default value of 0.9 σ a safe and advisable starting value, and it is recommended to keep this value below 1 σ.

sesize

In Figure 5, the raw accustomization image is depicted four times, each overlaid with the skeleton and nodes generated with a different value of `sesize`. The images shown are all at z = 230. The structural element are all spherical, with diameters of 0.5, 1.0, 2.0 and 2.5 σ for Figure 5A, B, C and D respectively. The remainder of the parameters are the default values.

Figure 5 shows that increasing `sesize` closes gaps with increasing size. This subroutine functions by the subsequent dilation (expansion of each material voxel by the structural element) and erosion (expansion of each non-material voxel by the structural element) of the image. With the structural element diameters of 0.5 σ, the smallest gap gets filled up. With increasing sizes, more and more gaps can get filled up. However, a too large element size leads to closing too many sections, which then leads to effects seen in Figure 5D, where the skeleton is at certain points missing. This is due to material being merged in the z-direction. As with the other two parameters, it is recommended to tune this parameter to the dataset that is being analyzed, with the default value of 0.5 σ an advisable starting value.

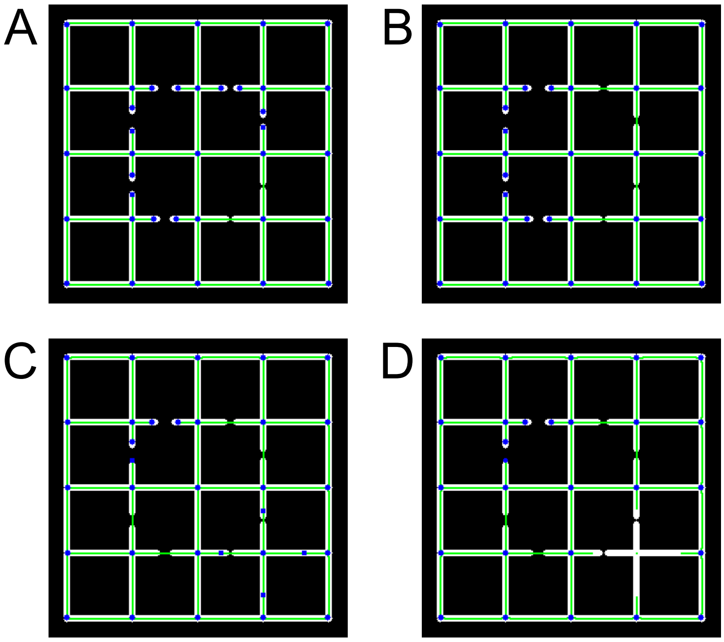

Figure 5: Overlays of ArGSLab's skeleton over the raw benchmarking image, using a different `sesize` but all other parameters default and constant. A) `sesize` = [0.5,0.5,0.5] σ, B) `sesize` = [1.0,1.0,1.0] σ, C) `sesize` = [2.0,2.0,2.0] σ, D) `sesize` = [2.5,2.5,2.5] σ.
